# Silver Nanoparticles Synthesized Using Wild Mushroom Show Potential Antimicrobial Activities against Food Borne Pathogens

**DOI:** 10.3390/molecules23030655

**Published:** 2018-03-14

**Authors:** Yugal Kishore Mohanta, Debasis Nayak, Kunal Biswas, Sameer Kumar Singdevsachan, Elsayed Fathi Abd_Allah, Abeer Hashem, Abdulaziz A. Alqarawi, Dhananjay Yadav, Tapan Kumar Mohanta

**Affiliations:** 1 Department of Botany, North Orissa University, Baripada 757003, Odisha, India; ykmohanta@gmail.com (Y.K.M.); sameer.bioteck@gmail.com (S.K.S.); 2 Department of Zoology, Seemanta Mahavidyalaya, Jharpokharia 757086, Odisha, India; deb63nayak@gmail.com; 3 Department of Biotechnology, Maulana Abul Kalam Azad University of Technology, Kolkata 700064, West Bengal, India; kunal.sapiens@gmail.com; 4 Plant Production Department, College of Food and Agriculture Science, King Saud University, Riyadh 11451, Saudi Arabia; eabdallah@ksu.edu.sa (E.F.A.A.); alqarawi@ksu.edu.sa (A.A.A.); 5 Department of Botany and Microbiology, College of Science, King Saud University, Riyadh 11451, Saudi Arabia; habeer@ksu.edu.sa; 6 Department of Medical Biotechnology, Yeungnam University, Gyeongsan 38541, Gyeongsangbuk-do, Korea; 7 Department of Biotechnology, Yeungnam University Gyeongsan, Gyeongsangbuk-do 38541, Korea

**Keywords:** silver nanoparticles, *Ganoderma sessiliforme*, antimicrobial activity, food borne bacteria

## Abstract

The present study demonstrates an economical and eco-friendly method for the synthesis of silver nanoparticles (AgNPs) using the wild mushroom *Ganoderma sessiliforme*. The synthesis of AgNPs was confirmed and the products characterized by UV-visible spectroscopy, dynamic light scattering spectroscopy and X-ray diffraction analysis. Furthermore, Fourier transform infrared spectroscopy (ATR-FTIR) analysis was performed to identify the viable biomolecules involved in the capping and active stabilization of AgNPs. Moreover, the average sizes and morphologies of AgNPs were analyzed by field emission scanning electron microscopy (FE-SEM). The potential impacts of AgNPs on food safety and control were evaluated by the antimicrobial activity of the synthesized AgNPs against common food-borne bacteria, namely, *Escherichia coli*, *Bacillus subtilis*, *Streptococcus faecalis*, *Listeria innocua* and *Micrococcus luteus*. The results of this study revealed that the synthesized AgNPs can be used to control the growth of food-borne pathogens and have potential application in the food packaging industry. Moreover, the AgNPs were evaluated for antioxidant activity (DPPH), for biocompatibility (L-929, normal fibroblast cells), and for cytotoxic effects on human breast adenosarcoma cells (MCF-7 & MDA-MB231) to highlight their potential for use in a variety of bio-applications.

## 1. Introduction

Nanotechnology, a rapidly growing field, has a variety of applications in biomedicine, food and engineering due to the exemplary characteristics of nanoparticles, such as biocompatibility, high productivity, speed of production, cost effectiveness and safety. The evolution of nanotechnology has been highly advantageous to mankind due to significant advances made using nanotechnology, such as advances in therapeutic applications [1], catalysis [2], bio-sensing devices and microelectronics [3], air and water purification, and paints [4]. Silver nanoparticles (AgNPs) are considered potent nano-weapons that can be used as active antimicrobial agents in the extermination of multidrug-resistant (MDR) bacteria [5]. Nanoparticles have enhanced catalytic activity due to their small size and large surface area [6]. AgNPs are routinely synthesized using physical, chemical and biological methods. However, the main disadvantages of the physical and chemical routes of synthesis are their higher operational costs, time and energy consuming steps. Further, their yield rate is relatively low with additional hazardous by–products possessing detrimental effects to the environment and human health [7]. Bio-based synthesis routes, on the other hand, provide various incentives such as wide variety of possible starting materials, and rapid and economic reaction rates with large scale production capabilities. The presence of various biologically active phytochemicals and metabolic compounds in biological samples eliminates the use of toxic chemicals for reducing or capping purposes during the reaction [8]. Various natural resources such as plants, plant products, and microorganisms (bacteria, fungi, algae, yeast and viruses), have been explored for the synthesis of metal nanoparticles [9].

Fungi are highly diversified microorganisms that have excellent abilities to produce novel secondary metabolites with potential application in different fields, such as therapeutics, bioremediation, bioleaching, and biocatalysis. Among the diverse fungi, *Aspergillus fumigatus* [10], *Fusarium oxysporum* [11] and *Verticillium* [12] are reported to form silver and gold nanoparticles. Additionally, rare fungi, such as *Penicillium fellutanum* from marine habitats, have also been reported to synthesize intracellular AgNPs [13]. The discovery of various novel fungal proteins having excellent reducing and capping properties has provided a platform for exploring novel fungal species in the biological synthesis of different metallic nanoparticles [14]. Mukherjee’s group reported that the aqueous extract of naturally occurring edible mushroom *Volvariella volvacea* acts as a reducing and capping agent during extracellular synthesis of Au, Ag and Au–Ag nanoparticles [15]. Recently our group has reported the synthesis of AgNPs using extracts of *Ganoderma lucidum* and *Ganoderma applanatum* both known for their medicinal properties exhibiting higher antimicrobial activities [16]. The aqueous extract of edible oyster mushrooms (*Pleurotus florida*) has also been shown to be a potential reducing agent during the photo-irradiated extracellular synthesis of silver nanoparticles [17].

*Ganoderma* sp. have been reported to exist throughout the world and more than 250 species of this mushroom are well documented [18]. *Ganoderma* sp. are classified into different categories according to their appearances and diverse color combinations which have distinct intrinsic biological properties. The group *Ganoderma* (*G.)*, including *G. lucidum*, *G. tsugae*, *G. applanatum* and *G. capense*, are commonly used in medicine [19]. Several comprehensive studies evaluating the pharmacological properties of *Ganoderma* sp. have revealed the antibacterial, antioxidant, anti-HIV, anti-inflammatory, antiproliferative, antidiabetic, anticancer, antitumour, hypocholesterolemic, hepatoprotective and other activities [20] of these bacteria. Mushrooms (macrofungi) are immensely wealthy in proteins and have been studied for their nutrient-richness as food stuffs and for their great medicinal value. However, very few reports have demonstrated the potential use of mushrooms for synthesis of metallic nanoparticles, which can be attributed to their seasonal appearance along with their abysmal location of growth. Therefore, the biological synthesis of AgNPs from mushroom extracts will provide a novel, simple, rapid, reliable, cost-effective and environmentally friendly approach for metallic nanoparticle synthesis. However, use of microorganism for synthesis of metallic nanoparticles provides some hindrance such as maintenance of aseptic culture conditions, chances of sample contamination and irregular size of produced nanoparticles [21,22].

In this era, there is an emerging convention of fast and pre-cooked food consumption due to busy lives; quick and easily cooked food materials are of great interest to human beings for sustaining their daily lives. Meanwhile, rapid food spoilage has become a major problem worldwide due to the conspicuous involvement of common harmful food-borne bacterial strains [23]. The evolution of multidrug resistant (MDR) bacteria with strong resistance to the currently available antibiotics is a major concern [24,25]. Due to their antagonistic effects these MDR bacteria are more pathogenic than their corresponding wild-type strains exhibiting higher rates of infection and mortality. Thus, constant search for new generation of disinfection strategies is highly necessary to counteract these bacterial pathogens [26]. Since food is life-sustaining a permanent solution for control of food spoilage and increased food safety is of immense importance. This can be achieved by exploring a new generation of antimicrobials and antioxidant agents. Due to advances in the use of nanotechnology in the field of antimicrobial research, silver nanoparticles have generated a great deal of interest as antimicrobial agent leads. In addition, AgNPs show potency against a broad spectrum of microbes at very low concentrations with almost negligible intrinsic toxicity towards other life forms [27]. AgNPs are approved and used for numerous biomedical and industrial applications, for example, as strong antimicrobial agents in medical devices, silicone rubber gaskets, textiles and fabrics and wastewater treatment plants [28]. Moreover, the consistent use of AgNPs in various food-related processes, such as agriculture, food manufacturing and packaging, can substantially reduce the population of pathogenic strains in food.

The present study was undertaken to explore the synthesis potential of the wild mushroom species *Ganoderma sessiliforme* for manufacturing AgNPs without utilizing any additional reducing or capping agents. The antimicrobial potential of AgNPs was evaluated against exemplary food-borne bacteria (*Escherichia coli*, *Bacillus subtilis*, *Streptococcus faecalis*, *Listeria innocua* and *Micrococcus luteus*) along with their antioxidant potential (DPPH assay). In vitro biocompatibility activity of AgNPs against (L-929) and anticancer activity of the produced AgNPs against two breast cancer cell lines (MCF-7 and MDA-MB 231) were also studied.

## 2. Results and Discussion

### 2.1. Biosynthesis and Characterization of AgNPs

**Figure 1:**
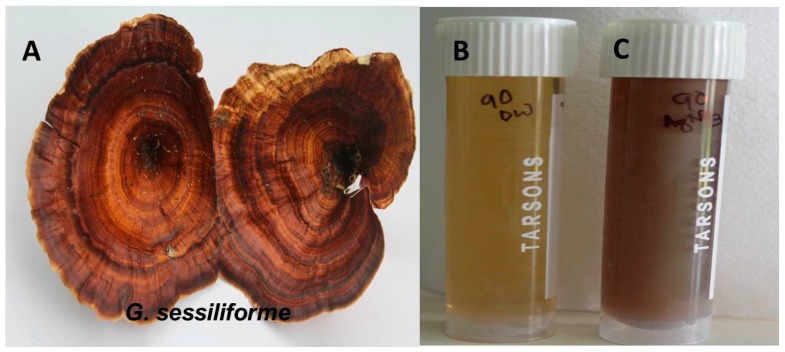
(**A**) *G. sessiliforme;* (**B**) *G. sessiliforme* mycelial extract and AgNO_3_; (**C**) synthesized silver nanoparticles (AgNPs).

The distinct color change of the aqueous AgNO_3_ solution within 60 min of incubation with the wild mushroom extract confirmed the rapid synthesis of AgNPs. The pale yellow-colored (AgNO_3_ + mushroom extract) solution changed to reddish brown, confirming the synthesis of AgNPs (Figure 1). The intensity of the reddish-brown color increased with time because of excitation due to surface plasmon resonance (SPR) and reduction of AgNO_3_.

UV–Vis spectrophotometry is a decisive and reproducible technique that is suitable for the accurate characterization of metal nanoparticles, though it has the drawback of not being able to rapidly provide information regarding particle charges and sizes. The sizes and shapes of the nanoparticles also influence the absorbance spectra (surface plasmon bands) as do the dielectric constants of the encompassing media. To investigate the rapid synthesis of nanoparticles various ratios of mushroom extract to AgNO_3_ was used. When 0.5:10 ratio was used the time taken for the change of color inference was very long, whereas the 1:10 ratio, took exactly 60 min for color change of the reaction mixture. Subsequently at 1.5:10 ratio, the reaction was very rapid but with formation of precipitate at the bottom of the flask. Hence, to further support the color change inference data subsequent UV spectroscopy analysis was conducted. It is presumed that smaller AgNPs can be synthesized by using lower concentrations of mushroom extract and precursor AgNO_3_. This concept has been demonstrated and reported by several researchers [29,30], and it was also confirmed in the present study by DLS and SEM analysis.

**Figure 2:**
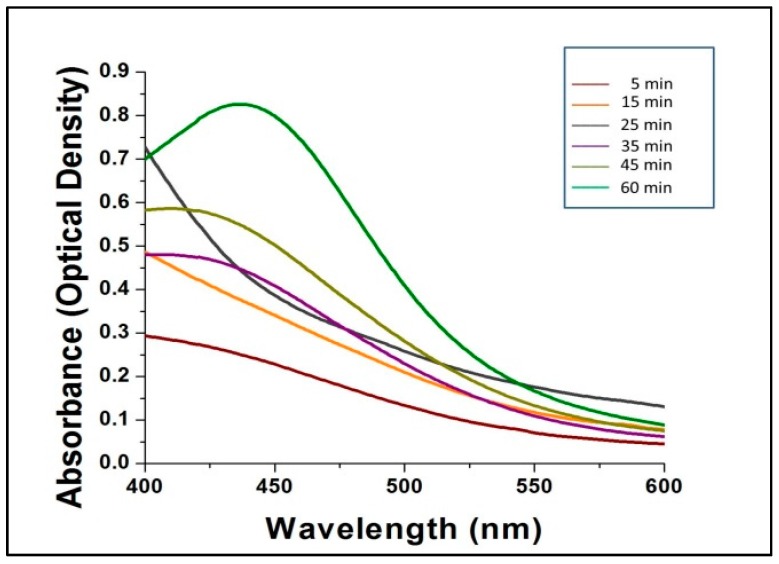
The ultraviolet-visible spectra of silver nanoparticles (AgNPs).

Figure 2 shows the UV-Vis spectra of the bio-synthesized AgNPs scanned at discrete time intervals. The reduction of AgNO_3_ by mushroom extract for the synthesis of AgNPs is expressed by the different colored absorption peaks at different time intervals (5, 15, 25, 35, 45 and 60 min) at room temperature (Figure 2). After 60 min, no further shifts in the spectrum were noted, which indicated complete consumption of the precursor AgNO_3_ in the reaction mixture, which gave a characteristic peak at 432 nm, indicating the synthesis of silver nanoparticles from AgNO_3_ by *G. sessiliforme* extract. There are several reports available regarding characteristic peaks shown by AgNPs in a spectral range of 430–450 nm [31,32,33,34,35].

**Figure 3:**
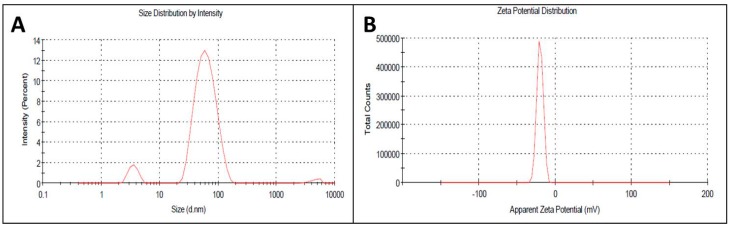
(**A**) Size distribution of synthesized AgNPs; (**B**) Zeta potential of synthesized AgNPs by DLS analysis.

The appropriate size and charge plays a major role in the structural and functional properties of any particle used in biological applications. In nanoscience, though the nano-size of each particle is appropriately small, several tiny nanoparticles are needed to improve activity, which they do by increasing the surface area. In the current investigation, the size and surface charge of the AgNPs were calculated by dynamic light scattering spectroscopy using an aqueous solution of AgNPs at ~45 nm and −19 mV (Figure 3A,B). At a 1:10 ratio of extract to AgNO_3_ ratio, the nanoparticles formed were of nanometer range, whereas at 1.5:10 ratio, large aggregates were formed with various size ranges in the size distribution profiles determined in the DLS studies. Hence, the UV spectroscopy and DLS study confirmed our 1st color change inference of nanoparticle synthesis at 1:10 ratio of the reaction mixture. AgNPs of uniform size and surface potential are used in various biological applications. The particle size is crucial for intracellular and intercellular transport, and a smaller size enhances the capacity of interaction of the particle with the protective cell membranes of the target organisms. Therefore, nano (<100 nm)-sized particles are used in drug delivery and as antimicrobial agents as well as in the development of biosensors [36,37]. In addition to the size, the surface charges of nanoparticles play a vital role in their interactions with biological macromolecules and biochemical pathways of living cells [38].

**Figure 4:**
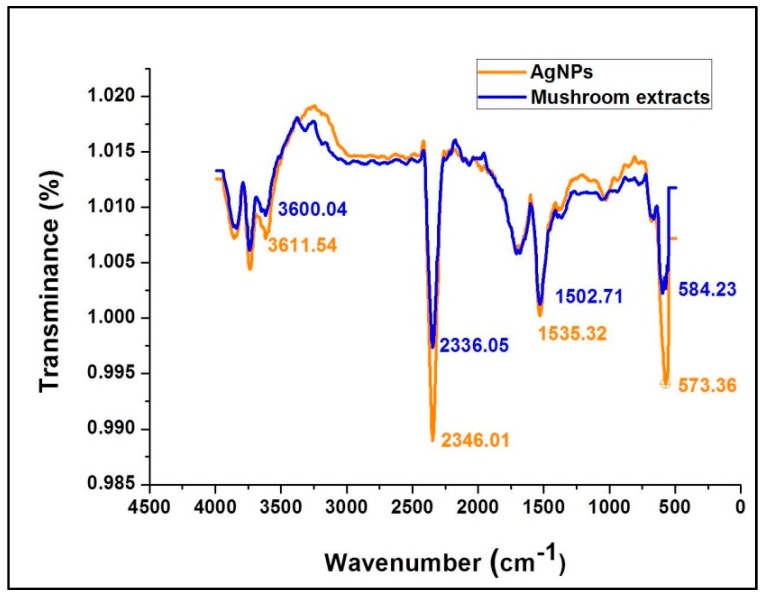
ATR-fourier-transformed infrared spectroscopy analysis of silver nanoparticles (AgNPs) and the aqueous extracts of *G. sessiliforme.*

ATR-FTIR spectroscopy provides specific background vibration and frequency analysis of compounds at the atomic level where specified chemical bonds represent a unique annotated molecular fingerprint for each compound. Figure 4 shows the FTIR spectral bands of the mushroom extract and silver nanoparticles synthesized using *G. sessiliforme* extract. Sharp transmittance peaks were observed at 3600.04, 2336.05, 1502.71, and 573.36 cm^−1^ in the mushroom extract and similar spectral bands were observed at 3611.54, 2346.01, 1535.32 and 573.36 cm^−1^ in freshly synthesized AgNPs. Upon comparison, similar transmittance bands were observed in both the mushroom extract and synthesized AgNPs. 

The occurrence of intense peaks at 3600.04 and 3611.54 cm^−1^ is indicative of O–H stretching which could be attributed to the presence of alcohol and phenolic groups in the mushroom extract which might get transferred to the AgNPs during the synthesis period. Another set of intense peaks were observed at 1502.71 and 1535.32 cm^−1^ which generally corresponded to N–O stretching that is associated with the presence of nitro [39]. From Figure 4 it can be clearly observed that functional groups associated with the mushroom extracts have imprinted their functional groups on the synthesized AgNPs. The prominent shifts in the positions of the distinct peaks in the AgNPs from the mushroom extract might be due to the reduction of AgNO_3_ by mushroom extracts with capping and stabilization of AgNPs by discrete mushroom secondary metabolites. Thus, from the FT-IR experiment in this study, it is evident that bioactive compounds identified as polyphenols, anthocyanins, flavonoids and nitro-compounds (proteins) present in *G. sessiliforme* play an integral role in the capping and stabilization of silver nanoparticles.

**Figure 5:**
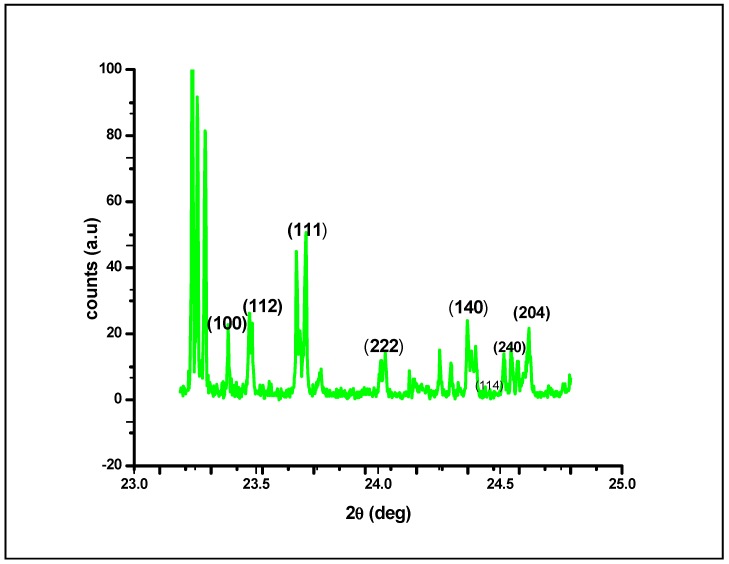
X–ray diffraction analysis of silver nanoparticles (AgNPs) synthesized by the aqueous extracts of *G. sessiliforme*.

The XRD plot shown in Figure 5 shows that at different diffraction angles, ranging from 23.5 to 25.0, different lattice planes of (100), (112), (111), (222), (140), (240), (114) and (204) exhibit various crystalline lattices. This variety in lattice planes could be attributed to the presence of irregular architecture in the native structure of AgNPs; upon irradiating the nanomaterial with X-rays, different lattice planes interacted with the X-rays, resulting in an X-ray diffraction plot of a typically crystalline nature at the nanoscale. By using the full width at half maximum (FWHM) values of the diffraction peaks and the Scherer equation, the average crystal size of the AgNPs was calculated as the mean value i.e., 45.26 nm. Figure 5 depicts four primary peaks with characteristic shifts of 222, 111, 112 and 100, which explains the crystalline structure of silver nanoparticles [40,41].

**Figure 6:**
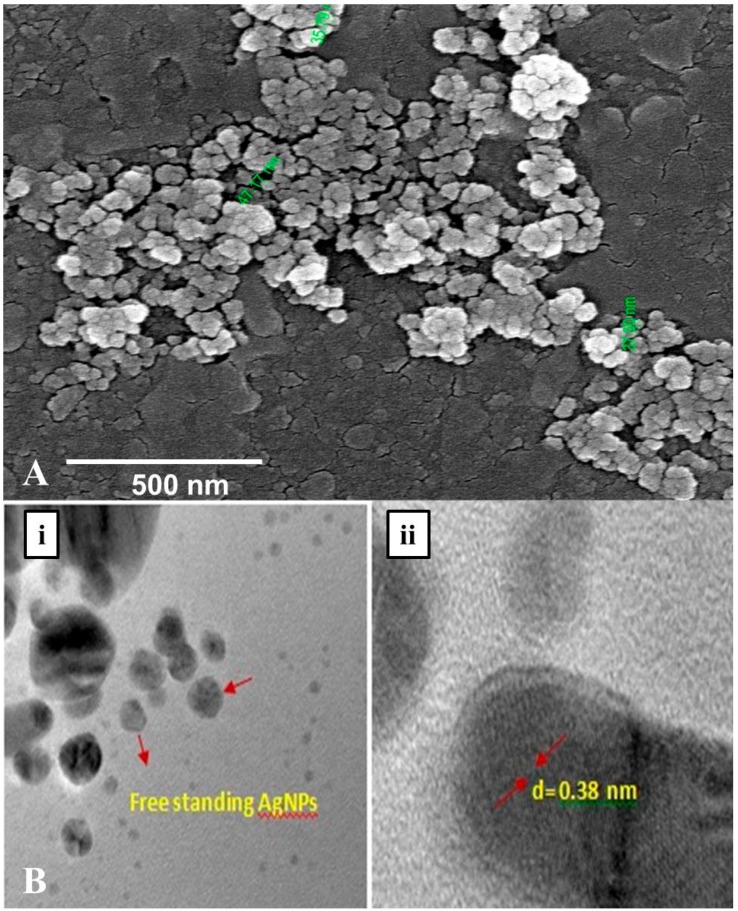
Morphological characterization through FE–SEM (**A**) and HR–TEM (**B**) microscopy of silver nanoparticles (AgNPs) synthesized by the aqueous extracts of *G. sessiliforme*; (i)Free standing AgNPs observed in HR–TEM; (ii) analysis of grain diameter of a single AgNP in HR–TEM.

The morphology along with the spherical shape and polydisperse nature of AgNPs capped with its bio–moieties were confirmed by the FE-SEM and HR–TEM micrograph (Figure 6A,B). The FE-SEM and HR–TEM micrographs reflect the regular, spherical shape of AgNPs with smooth edges having average size of ~45 nm. Further, in the TEM images (Figure 6B) it is clearly visible that our synthesized nanoparticles are not agglomerated and are freely scattered which is a very important property for drug delivery. Interestingly in DLS studies also approximately 70% of the scanned samples exhibited size of ~45 nm during scanning. Hence, from the dynamic light scattering studies, Fe–SEM and HR–TEM micrographic studies we conform that our *G. sessiliforme* extract mediated synthesized AgNPs were in the nano-range with roughly spherical morphology. Due to this shape and size, the nanoparticles could have a profound impact on drug conjugation and cell targeting during drug delivery [42].

### 2.2. Qualitative and Quantitative Assessments of Phytochemicals and DPPH-Scavenging Activities

Results from the qualitative and quantitative phytochemical assessments of the aqueous *G. sessiliforme* extracts have been compiled in Table 1 and Table 2. The phytochemical study shows the presence of flavonoids, phenolics, proteins, tannins and sugar, whereas there is a complete absence of glycosides, steroids and sterols. The phytochemical evaluation of the aqueous *G. sessiliforme* extracts indicated the involvement of flavonoids, tannins, phenolic compounds, sugars and proteins as principal chemical constituents in the synthesis of AgNPs. There are very few reports that provide details about the involvement of these mushroom molecules in AgNP synthesis. Karwa et al. [42] reported the active participation of amide and protein linkages in the stable synthesis of AgNPs [43]. A predicted mechanism of AgNP biosynthesis hypothesized about the involvement of antioxidant enzyme complexes in the biosynthesis of silver nanoparticles [44]. Moreover, antioxidant activity is strongly attributed to the presence of flavonoids and phenolic compounds in the extracts of plants or mushroom [14]. From such reports and analyses, the active contribution of flavonoids, phenolics and sugars in the biosynthesis process is evident.

**Figure 7:**
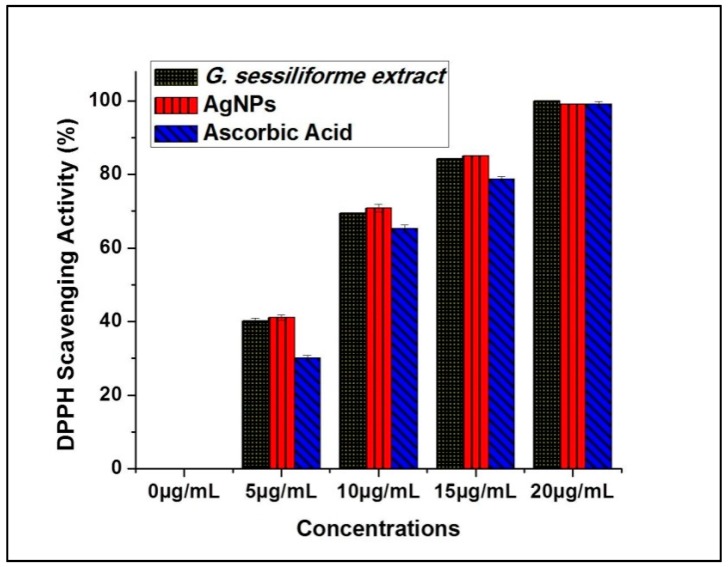
Antioxidant potentials of AgNPs and ascorbic acid (DPPH radical scavenging).

Antioxidant potential (DPPH radical scavenging activity) measurements for *G. sessiliforme* extract clearly suggest the probable contribution of antioxidant molecules from the mushroom extract to the biosynthesis of silver nanoparticles. The large stores of phenolics and flavonoids in *G. sessiliforme* lead to high antioxidative efficiency, and these compounds are considered powerful free-radical scavengers. Further, since such molecules are the core molecules responsible for capping and stabilizing the AgNPs, the nanoparticles are also expected to have antioxidant potential, which can be very useful for the biomedical industry. Significant antioxidant efficacy was also observed by DPPH assays in both *G. sessiliforme* extracts and AgNPs (Figure 7).

The antioxidant (DPPH scavenging) capacity was found to be (IC_50_ = 6.19 ± 0.05 µg/mL) in *G. sessiliforme* extracts and (IC_50_ = 6.10 ± 0.04 µg/mL) in AgNPs. The presence of phenolics and flavonoids in *G. sessiliforme* indicated a high antioxidant activity. The high molecular weight molecules, along with the proximity and abundance of aromatic rings and hydroxyl groups present in bioactive compounds, have a significant effect on free radical scavenging activity [45].

It is important to demonstrate the antioxidant potential of AgNPs since some of the biomolecules (capping and stabilizing agents) from the mushroom extracts are still present in the AgNPs after purification and may have adverse effects on living cells. Thus, the antioxidant potential of *G. sessiliforme* is involved in the biological synthesis process of AgNPs and can be considered to be extremely safe for biological applications.

### 2.3. Biological Activity

#### 2.3.1. Antibacterial Activity

**Figure 8:**
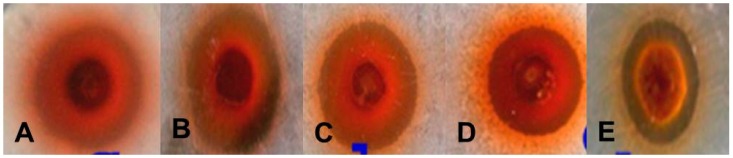
Antibacterial activities of AgNPs synthesized by *G. sessiliforme* (volume-50 µL/well): (**A**) *M. luteus*; (**B**) *L. innocua*; (**C**) *B. subtilis*; (**D**) *S. faecalis*; (**E**) *E. coli*.

Preliminary results on the antibacterial activity of AgNPs were obtained by screening *Escherichia coli*, *Bacillus subtilis*, *Streptococcus faecalis*, *Listeria innocua* and *Micrococcus luteus* using the agar well diffusion method (Table 3). A zone of inhibition was found against all gram-positive and gram-negative bacteria (Figure 8). After screening by the agar well diffusion method, the antimicrobial activity was confirmed by a micro-broth dilution assay and the percentage (%) of inhibition and the minimum inhibitory concentration (MIC) of each strain were determined (Table 4). Four gram-positive strains exhibited growth inhibition above 90% whereas gram-negative *E. coli* exhibited growth inhibition below 90%. The MICs for all strains were calculated in terms of IC_50_ values (Table 4).

Healthcare systems worldwide face tremendous destruction due to the development of drug-resistant pathogenic strains [46]. Thus, the potential antimicrobial effects of AgNPs on food-borne pathogenic bacteria (Table 3) represent a step towards the formulation of new potential antibacterial drugs. As silver shows very little or negligible toxicity towards animal cells but exhibits high toxicity against microorganisms. AgNPs could be beneficial in the development of antimicrobial agents or drugs to eradicate bacterial pathogens.

Though there are numerous reports on the antibacterial activity of silver nanoparticles, the exact mechanism of the antimicrobial activity of AgNPs against pathogenic bacteria remains unclear. Sondi and Salopek-Sondi have hypothesized about the involvement of physical entities in the antimicrobial mechanism. Studies have revealed that the electrostatic forces between the positively charged AgNPs and negatively charged bacterial cells may be responsible for the bactericidal effects of AgNPs [47]. The positive antimicrobial activity of silver nanoparticles can be attributed to several possible mechanisms, such as the disruption of metabolic enzymes, inactivation of cellular proteins and breakage of DNA [28,48]. As we further develop these nanoparticles, due to their novel small size and high surface area, AgNPs might have tremendous potential to inactivate common functions or metabolic processes, such as permeability, respiration, and energy generation, in pathogenic bacteria. In addition, the inability of microbes to fight against nanoparticles allows AgNPs to easily invade the inner cell components and cause severe damage to the cells by interacting with sulfur- and phosphorus-containing compounds, such as proteins and genetic materials, which leads to complete cell lysis [28,49,50,51,52,53]. Such antimicrobial properties of AgNPs are promising for the development of potential antimicrobial agents or drugs for industrial application, e.g., the formulation of polymeric packaging materials for foodstuffs and other durable polymeric materials, which could be resistant to harmful microorganisms [54,55].

#### 2.3.2. Biocompatibility and Anticancer Activity Study

**Figure 9:**
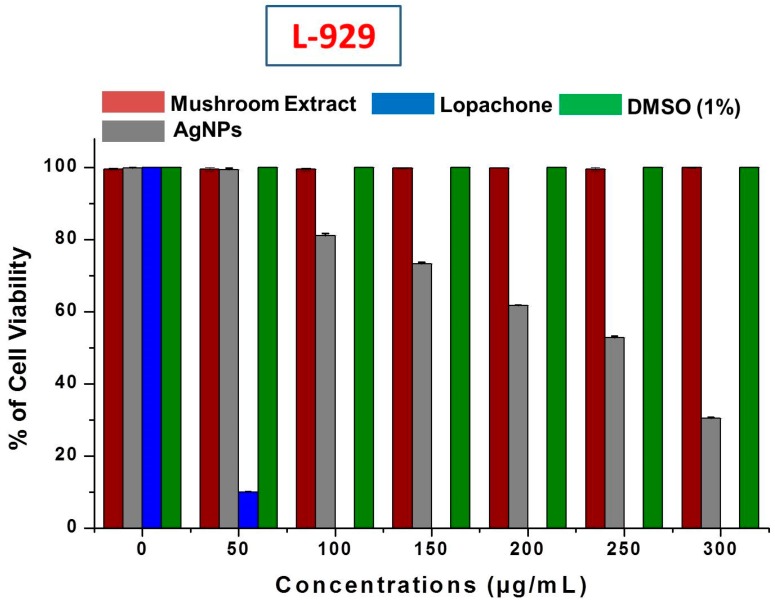
Cytotoxic effect of silver nanoparticles (AgNPs) on L-929 normal fibroblast cell lines.

It is very important to study the biocompatibility of silver nanoparticles for their successful implementation as a formulated product, for example, in drugs, and for their supplemental use in human consumption as food additives. Therefore, the cytotoxicity of AgNPs was evaluated against L-929 normal fibroblast cell lines. When using AgNPs with food materials, their safety and toxicity towards normal cell lines is a major concern. This study revealed that no inhibition or toxicity of biosynthesized AgNPs in the L-929 cell line was observed at low AgNPs concentrations. However, the cell viability decreased with increasing AgNPs concentrations (Figure 9). The IC_50_ of AgNPs was calculated as 256.6 ± 0.92 μg/mL against normal L-929 cell lines. The IC_50_ results strongly suggested that AgNPs obtained using mushroom extracts are biocompatible and safe for use in the food industry for developing value-added products. Further, the mushroom extract has no significant toxicity against the L-929 cell line, which allows the use of wild mushroom extract as bioreducing agents in the biosynthesis of AgNPs. Our results support a previous study of the biocompatibility of AgNPs with Chinese hamster ovary (CHO) cells [56].

**Figure 10:**
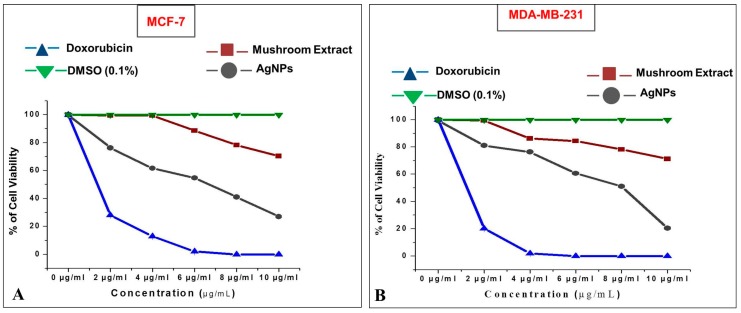
Cytotoxic effect of silver nanoparticles (AgNPs) on: (**A**) MCF-7; (**B**) MDA-MB-231 human breast cancer cells.

In addition to the biocompatibility study, the anticarcinogenic property of AgNPs was evaluated against human breast adenocarcinoma (MCF-7 and MDA-MB-231) cells. The mushroom-mediated AgNPs showed good anticarcinogenic activity against both the breast cancer cell lines with an IC_50_ of 6.62 ± 0.05 μg/mL against MCF-7 (Figure 10A) and 8.06 ± 0.01 μg/mL against MDA-MB-231 (Figure 10B).

The inhibition of the proliferation of human glioblastoma cells by AgNPs has been previously studied [57]. In addition to our current study, Franco-Molina et al., have also evaluated the impact of colloidal Ag on human breast cancer cells (MCF-7) [58]. The exact metabolic activities of AgNPs in killing cancer cells are very rarely studied. The mechanistic study by Sanpui et al. revealed that AgNPs generally disrupt the normal cellular function, interfere with membrane integrity and induce numerous apoptotic signaling genes of mammalian cells which leads to programmed cell death [59]. Hsin et al. [59] reported the induction of apoptosis in NIH3T3 (murine embryonic fibroblasts) cells by heightening reactive oxygen species (ROS) generation by AgNPs. Moreover, the AgNPs are highly responsible for mitochondria-dependent apoptosis by activation of the c-Jun N-terminal kinase pathway. Gurunathan et al. performed a detailed investigation of the cytotoxic effect of mushroom (*Ganoderma neo-japonicum*)-mediated AgNPs on the breast cancer cell line MDA-MB-231 [60]. Their study showed apoptotic cell death of MDA-MB231 cells due to the activation of caspase 3 and nuclear DNA fragmentation. Moreover, the cell death was due to increased reactive oxygen species and hydroxyl radical production upon exposure to AgNPs. The anticancer activity of *G. sessiliforme*-mediated AgNPs demonstrates its potential as an alternative candidate in the treatment of breast cancer.

## 3. Materials and Methods

### 3.1. Sample Preparation

The wild mushroom *Ganoderma sessiliforme* was collected from the periphery of the Similipal Biosphere Reserve (Odisha, India). The mushroom samples were identified on the basis of their macroscopic and microscopic characteristics using a standard identification manual [61]. Fine dried mushroom powder (10 g) was combined with Milli-Q water (100 mL, followed by sonication for 20–25 min. The sonicated aqueous mushroom extract was clarified by repeated centrifugation at 4000 rpm. After centrifugation, the extract was filtered through filter paper (Whatman No. 40) and stored at 4 °C until further use.

### 3.2. Biosynthesis of Silver Nanoparticles (AgNPs)

The synthesis of AgNPs was conducted using *G. sessiliforme* extract adopting a green synthesis route. Briefly, mushroom extract (10 mL) was combined with 1 mM AgNO_3_ (100 mL) in a conical flask and agitated continuously at room temperature till the appearance of a reddish-brown color. Various ratios of mushroom extract to AgNO_3_ (0.5:1; 1:10; 1.5:10) were used during the synthesis for getting the ideal shape and size of AgNPs.

### 3.3. Characterization of Silver Nanoparticles

The freshly synthesized AgNPs were characterized by UV-Vis spectroscopy, dynamic light scattering spectroscopy (DLS), high-resolution transmission electron microscopy (HR-TEM), attenuated total reflection Fourier transform infrared spectroscopy (ATR-FTIR), and X-ray powder diffraction (XRD) following standard analytical procedures. The biosynthesis of the AgNPs was continuously monitored by UV-Vis spectroscopy by calibrating the absorption spectra (350–600 nm) at a resolution of 1 nm (Lambda 35^®^, Perkin Elmer, Waltham, MA, USA). The surface charge, polydispersity index (PDI) and hydrodynamic radius, of the synthesized AgNPs were carried out using a Zeta Sizer (ZS 90, Malvern Instruments Ltd., Malvern, UK). The nano-dimensional structures of Ag particles were confirmed by field emission scanning electron microscopy (Nova, NanoSEM, 450/FEI, FEI, Lincoln, NE, USA) performed at acceleration voltage of 15 kV. HR–Transmission Electron Microscopy (Technai™ F30 G^2^ STWIN, FEI, Lincoln, NE, USA) was used. The nanoparticles were drop coated in a copper grid with mesh size 300 and were observed at 300 kV. The possible role of the mushroom metabolites on surface modification of the AgNPs was analyzed using an attenuated total reflection Fourier transform infrared spectrophotometer (Bruker ALPHA, Ettlinger, Germany) at a resolution of 4 cm^−1^. The silver nanoparticles were studied in a range of spectral regions (4000–500 cm^−1^) with an average of 25 scans per sample, and the results obtained were analyzed by OPUS software. The crystalline properties of AgNPs were analyzed using an X-ray diffractometer (PANalyticalX’Pert, Almelo, The Netherlands) equipped with a Ni filter and a Cu Kα (l = 1.54056 Å) radiation source. The scanning rate was 0.05 degrees, while the diffraction angle varied in the range of 20–80 degrees.

### 3.4. Biological Activities

#### 3.4.1. Antibacterial Activity

Promising antibacterial activity of AgNPs was observed against the common food-borne bacteria *Escherichia coli*, *Bacillus subtilis*, *Streptococcus faecalis*, *Listeria innocua* and *Micrococcus luteus* by the standard agar well diffusion and micro-broth dilution methods described in our previous study [62]. The bacterial pathogens were procured from the Microbial Type Culture Collection (MTCC, Chandigarh, India) and nutrient agar media (HiMedia, Mumbai, India) was used to maintain the strains. Prior to the antibacterial study, a colloidal suspension of the AgNPs was made by suspending AgNPs in 5% dimethyl sulfoxide (DMSO) and sonicating for 15–20 min at 30 °C. To evaluate the antibacterial activity, 100 μL (MHB) of each test organism were implanted on the MHA plates followed by the preparation of wells that were 5 mm in diameter and 2.5 mm deep. Fifty microliters of the AgNP (1 mg/mL) suspension was poured into the well. A standard antibiotic, kanamycin (5 mg/mL), was used as a positive control, while 5% DMSO was used as a negative control. The antibacterial efficacy of the AgNPs was estimated by calculating the inhibition zone after 24 h of incubation at 37 °C. Furthermore, a micro-broth dilution method was adopted to confirm antibacterial activity and to determine the minimum inhibitory concentration (MIC) of AgNPs as described in a previous study. A 90% inhibition capacity was considered promising and subsequent experiments were conducted to determine the MIC values. The MIC was determined and expressed as IC_50_ using IC_50_/IC_90_ Laboratory Excel Calculation Tools. Each experiment was carried out thrice and zones (%) of inhibition were presented as mean ± standard deviations (SD).

#### 3.4.2. Qualitative Phytochemical Analysis

A qualitative phytochemical investigation of wild mushroom *G. sessiliforme* extract was executed using a standard method [63,64]. The required chemicals and reagents used for the qualitative phytochemical analysis were procured from Sigma-Aldrich, Mumbai, India). The results obtained from the experiments were designated positive (+) or negative (–) [65].

### 3.5. Quantitative Phytochemical Analysis and In Vitro Antioxidant Properties

#### 3.5.1. Total Phenolic Content (TPC) Determination

The Folin-Ciocalteu method [66] was adopted to determine the total phenolic content (TPC) of the mushroom extract, and the results were expressed as gallic acid equivalents (GAE) in mg/g sample. All the experiments were conducted in triplicate.

#### 3.5.2. Total Flavonoid Content (TFC) Determination

A modified standard aluminum chloride method was employed to determine the total amount of flavonoids (TFC) and the results were expressed as gallic acid equivalents (GAE) in mg/g sample. All estimations were performed in triplicate.

#### 3.5.3. 1,1-Diphenyl-2-picrylhydrazyl (DPPH) Radical Scavenging Activity

The most common and preferred method, namely, the 1,1-diphenyl-2-picrylhydrazyl (DPPH) assay, was adopted to determine the potential antioxidant activity [66]. Different concentrations (5, 10, 15 and 20 µg/mL) of AgNPs were chosen for the study of DPPH scavenging capacity. The MIC for DPPH scavenging was determined, and the radical scavenging activities (%) were expressed as IC_50_ values. Ascorbic acid (equivalent concentrations of AgNPs) was used as a positive control.

### 3.6. In Vitro Biocompatibility and Anticancer Activity

#### 3.6.1. Cell Line Culture and Treatment of AgNPs

Normal fibroblast cell line (L-929) and human breast cancer MCF-7 and MDA-MB 231 cell lines were procured from the NCCS (Pune, India). The cells were cultured under standard conditions in Dulbecco’s Modified Eagle’s Medium (DMEM) (Sigma–Aldrich, Mumbai, India) enriched with 10% FBS and M-199 medium, penicillin (100 U/mL), and streptomycin (100 U/mL) with incubation at 37 °C in a 5% CO_2_ atmosphere [67]. After incubation, the surface-affixed cells were thoroughly trypsinized for 3–4 min and centrifuged (750 rpm, 8–10 min) to obtain individual cells. After centrifugation, the cells were counted and distributed in a microplate (96 well) at 5000 cells/well and incubated for 24 h (37 °C, 5% CO_2_) to form a confluent (~70–80%) monolayer [57]. The toxicities of the AgNPs against the L-929, MCF-7 and MDA-MB 231 cell lines were determined at increasing concentrations (10–250 μg/mL); the experiment was performed in triplicate and the populations of the cells were counted (2030 Multilabel Processor VictorTMX3 Perkin Elmer, Waltham, MA, USA) at 48 h.

#### 3.6.2. Cell Viability Study by MTT Assay

The cell viability was determined by adding MTT solution (200 μL) to each culture well and incubating for 4–5 h followed by the removal of MTT solution and addition of DMSO (200 μL) to each well and incubation for 15 min in the dark. Subsequently, the optical density of the formazan product was measured at 595 nm in a microplate reader (2030 Multilabel Processor VictorTMX3, Perkin Elmer, Waltham, MA, USA) [68].

### 3.7. Statistical Analysis

The results of all the experiments were presented as the mean value of three independent replicates with standard deviations (SD). Statistical analysis of the significant differences between the mean values of the results was performed by one-way analysis of variance (ANOVA) followed by Duncan’s test at a 5% level of significance (*p* < 0.05).

## 4. Conclusions

The successful synthesis of AgNPs by active reduction of silver ions by using wild mushroom *G. sessiliforme* extracts as bioreductant developed an imperative new green technology solution, where mushrooms could be used actively for metal nanoparticles synthesis. The potential antimicrobial activity against food borne bacteria will be boon for the food industry to use such metal nanoparticles to reduce the contamination of food stuffs and also for long time preservation. The major complication of food industry such as food contamination and spoilage by bacteria now can be resolved with continuous effort of using metal nanoparticles. The cytotoxicity results indicated that the silver nanoparticle has potential anticancer activity in breast cancer cell lines, suggesting that silver nanoparticle might be a possible alternative promoter for human breast cancer therapy. Moreover, the biocompability against normal fibroblast cell lines strongly suggested of complete safe use of biosynthesized silver nanoparticles for food related industry and assistant agents during preparation of food materials and packaging.

## Figures and Tables

**Table 1:** Qualitative phytochemical screening of aqueous extract of *G. sessiliforme*.

Phytoconstituent	Observation
Alkaloids	−
Tannins and phenolic compounds	+++
Glycoside	−
Flavonoids	+++
Steroids and sterols	−
Triterpenoids	+
Sugars	+++
Proteins	+++

Notes: +++: Highly present; +: Less present; −: Absent.

**Table 2:** Quantitative phytochemical constituents of aqueous extract of *G. sessiliforme*.

Phytochemical Constituent	mg/100 g Dry Weight (Mean ± SD)
TPC	620.67 ± 28.00
TFC	845.26 ± 24.20

**Table 3:** Antimicrobial activity of AgNPs by agar-well diffusion method.

Mean Zone of Inhibition ± SD (in mm)			
Name of The Test Strain	Silver Nanoparticles (500 µg/mL)	Kanamycin (5 mg/mL)	DMSO (5%)
*Escherichia coli*	11 ± 0.50	20.8 ± 0.59	0
*Bacillus subtilis*	20 ± 1.00	13.3 ± 0.12	0
*Streptococcus faecalis*	16 ± 1.00	11.1 ± 0.13	0
*Listeria innocua*	22 ± 1.15	12.3 ± 0.21	0
*Micrococcus luteus*	21 ± 1.15	10.2 ± 0.31	0

**Table 4:** Antimicrobial activity of AgNPs by micro broth dilution method.

Antibacterial Activity of AgNPs (Percentage of Inhibition (%) ± SD)						
Name of The Test Strain	1000 µg/mL	500 µg/mL	250 µg/mL	125 µg/mL	61.25 µg/mL	IC_50_ (µg/mL)
*Escherichia coli*	83.10 ± 0.08^a^	62.47 ± 0.26^b^	40.33 ± 0.21^c^	36.33 ± 0.09^d^	35.33 ± 0.09^e^	338.39 ± 1.71
*Bacillus subtilis*	99.53 ± 0.29^a^	99.44 ± 0.26^a^	99.13 ± 0.17^a^	71.47 ± 0.21^b^	20.37 ± 1.33^c^	93.38 ± 0.70
*Streptococcus faecalis*	94.63 ± 0.39^a^	92.80 ± 0.24^b^	60.90 ± 0.22^c^	46.03 ± 0.12^d^	34.67 ± 0.37^e^	150.40 ± 1.00
*Listeria innocua*	93.93 ± 0.87^a^	94.17 ± 0.12^a^	80.20 ± 0.22^b^	60.20 ± 0.08^c^	35.03 ± 0.25^d^	94.38 ± 0.25
*Micrococcus luteus*	94.27 ± 0.12^a^	93.70 ± 0.22^b^	70.27 ± 0.12^c^	55.33 ± 0.08^d^	32.30 ± 0.16^e^	106.55 ± 0.32

The data are expressed as a percentage inhibition of bacteria and represent the mean ± SD (*n* = 3). Antimicrobial activity: exponentially growing cells were treated with different concentrations of AgNPs for 24 h and cell growth inhibition was analyzed through broth dilution assay. In each row, mean values followed with different superscripts significantly differ from each other according to Duncan’s Multiple Range Test (*p* < 0.05). IC_50_ is defined as the concentration, which results in a 50% reduction in cell numbers as compared with that of the control cultures (AgNPs). The values represent the mean ± SD of three individual observations.
